# Computer vision–guided open-source active commutator for neural imaging in freely behaving animals

**DOI:** 10.1117/1.NPh.11.3.034312

**Published:** 2024-09-26

**Authors:** Ibrahim Oladepo, Kapil Saxena, Daniel Surinach, Malachi Lehman, Suhasa B. Kodandaramaiah

**Affiliations:** a University of Minnesota, Twin Cities, Department of Mechanical Engineering, Minneapolis, Minnesota, United States; b University of Minnesota, Twin Cities, Department of Biomedical Engineering, Minneapolis, Minnesota, United States; c University of Minnesota, Twin Cities, Graduate Program in Neuroscience, Minneapolis, Minnesota, United States

**Keywords:** calcium imaging, computer vision, miniaturized neurophotonics

## Abstract

**Significance:**

Recently developed miniaturized neural recording devices that can monitor and perturb neural activity in freely behaving animals have significantly expanded our knowledge of neural underpinning of complex behaviors. Most miniaturized neural interfaces require a wired connection for external power and data acquisition systems. The wires are required to be commutated through a slip ring to accommodate for twisting of the wire or tether and alleviate torsional stresses. The increased trend toward long-term continuous neural recordings has spurred efforts to realize active commutators that can sense the torsional stress and actively rotate the slip ring to alleviate torsional stresses. Current solutions however require the addition of sensing modules.

**Aim:**

Here, we report on an active translating commutator that uses computer vision (CV) algorithms on behavioral imaging videos captured during the experiment to track the animal’s position and heading direction in real time and uses this information to control the translation and rotation of a slip ring commutator to accommodate for accumulated mouse heading orientation changes and position.

**Approach:**

The CV-guided active commutator has been extensively tested in three separate behavioral contexts.

**Results:**

We show reliable cortex-wide imaging in a mouse in an open field with a miniaturized wide-field cortical imaging device. Active commutation resulted in no changes to measured neurophysiological signals.

**Conclusion:**

The active commutator is fully open source, can be assembled using readily available off-the-shelf components, and is compatible with a wide variety of miniaturized neurophotonic and neurophysiology devices.

## 1 Introduction

Understanding how the brain mediates complex behaviors requires the synchronized acquisition of both large-scale neural activity and behavior monitoring. Miniaturized neural devices that can be docked to mice, for neural recording and active manipulation of activity, have become key tools for neuroscience to address these questions.

Miniaturized neurophotonic imaging devices allow imaging of specific populations of neurons at a large scale and have been particularly instrumental in conducting these critical neuroscience studies.^1^^–^^3^ The generally open-source culture underpinning these innovations has spurred the development of myriad miniaturized neurophotonic with new variants of miniscopes that have smaller form factors and multiple Field of views (FOVs), and^4^^,^^5^ large FOVs.^1^^,^^6^ Variants that incorporate optics for structured optogenetic stimulation^7^ and simultaneous electrophysiology recording^8^ have also been developed. These tools complement the head-borne electrophysiology recording devices, such as tetrode microdrive,^9^^–^^14^ and more recent miniaturized high-density complementary metal oxide semiconductor (CMOS) recording probes.^15^^–^^19^

Most miniaturized head-borne devices require a wired tether for powering the devices and interfacing with a fixed remote data acquisition system. The wire can get entangled as behaving mice locomote naturally in the behavior arenas or when they perform tasks requiring them to execute several stereotyped behavioral trajectories (such as in an eight-maze choice task^20^). This is typically mitigated using passive commutators that can relieve the torsion in the wires and transfer data through a slip ring. The increased trend toward long-term continuous neural recordings has spurred efforts to realize active commutators. These active commutators rely on inertial sensing, and both these solutions require the incorporation of additional hardware elements within the head-borne devices, which constrains the design space where functional elements of the head-borne device need to be limited to <3  g (∼15% of bodyweight of a 20-g mouse). Wireless data transfer rates are typically limited and further require specialized hardware surrounding the behavioral arena for powering and data transfer.

To mitigate this issue, active commutator systems utilizing inertial measurement unit (IMU),^21^^,^^22^ magnetic rotation sensor,^23^ torque sensor,^14^ Hall sensor,^24^^,^^25^ and video-based tracking^26^ have been developed. Measurement of an animal’s heading angle using a torque sensor to estimate animal rotations (by indirectly measuring tethered cable rotations) is easy to implement, but the effectiveness of this approach is limited by sensitivity issues, as cables are poor transmitters of torque.^14^^,^^21^ Similarly, Hall sensor and magnetic rotation sensor-based heading angle estimation approaches are affected by sensitivity. The IMU-based heading angle estimation approach is not affected by the cable sensitivity issue but requires calibration due to magnetic distortions.^21^ Further, the IMU data can necessitate adding more channels to the commutator for data transfer, adding more weight to the already limited payload and limited cable data transfer bandwidth.

Video-based methods offer potential solutions to issues with torque or inertial sensing and have been explored previously where light-emitting diodes (LEDs) on the head-mounted devices could track the position and direction of a mouse in the captured video frames.^26^ The incorporation of LEDs however requires modification to existing miniature imaging and recording technologies. Given that monitoring the behavior of the animal is a critical requirement, and in these experiments, making use of this information to actively rotate a commutator and further move the commutator along with a mouse across a large behavior arena could enable new kinds of experiments with existing miniaturized devices.

Here, we present a computer-vision (CV)–guided active translating commutator. The CV-guided active commutator leverages recent advances in real-time markerless tracking to estimate the location and heading direction of the animal and uses a translating stage and a motor to move and rotate a slip ring commutator along with the mouse. We show that position and heading direction can be computed in real-time at 6 Hz in three different behavioral assays—open field behavior, active place avoidance behavior, and the Barnes maze spatial navigation task. We further show that this information can be used to actively control the position and angular orientation of the slip ring commutator in response to mouse locomotion in the active place avoidance (APA) task and exploration of a linear maze.

## 2 Methods

### 2.1 General Design of the CV-Guided Active Commutator

**Fig. 1:**
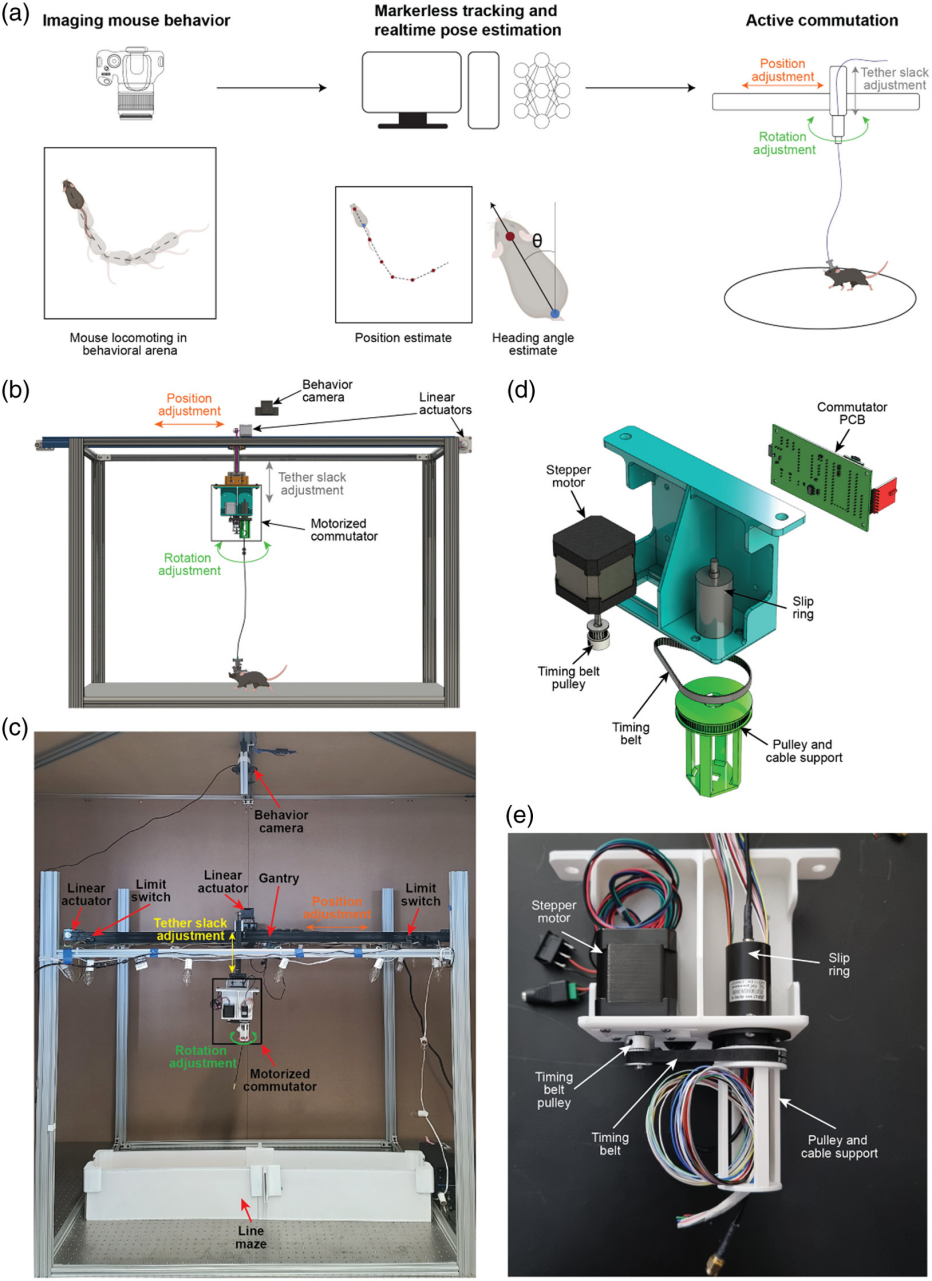
Open-source CV-guided active, translating commutator system. (a) Principle of operation—mouse behavior video captured from an overhead camera is used to estimate the real-time position of the mouse location and heading direction, which is used to actively translate and rotate a slip ring commutator. (b) CAD schematic of the CV-guided active translating commutator system. (c) Photo of the CV-guided active translating commutator system. (d) Detailed CAD schematic of the motorized commutator module. (e) Photograph of the motorized commutator module.

The CV-guided active commutator system dynamically adjusts the position and angular orientation of a slip ring commutator in response to the estimated movement and angular orientation of a mouse within a behavioral arena. The overall principle of operation is illustrated in Fig. 1(a). An overhead camera captures video of the mouse behaving in the arena. A deep neural net algorithm [DeepLabCut (DLC)^27^] is used to estimate the real-time position and heading angle direction of the mouse. These estimates of the mouse position and angular heading direction are used to actively translate and rotate a slip ring commutator.

The overall architecture of the CV-guided active commutator hardware is shown in Fig. 1(b). The CV-guided commutator consists of two main components, a behavior imaging camera located above an arena gantry that supports the actuator components. Two stepper motors (NEMA 17, OpenBuilds Parts Store, Zephyrhills, Florida, United States) coupled to linear actuator modules provide actuation in x and z directions. A rotation module is mounted on the z-translation stage [Figs. 1(b) and 1(c)]. A third stepper motor (NEMA 17) is coupled to the slip ring commutator via a timing belt drive [Fig. 1(d)]. We tested two slip rings: a single-channel coax slip ring with 24 channel plane wires (LPC-24YT-2402-01HC, JINPAT Electronics, Shenzhen, China) and a three-channel universal serial bus (USB) slip ring with 24 plane wires (LPT000-2402-04HF, JINPAT Electronics). The stepper motor and slip ring were attached to the anterior side of the three-dimensional (3D)-printed holding structure. A custom printed circuit board (PCB) was designed to wirelessly receive commands from a computer and drive the stepper motor (Fig. S1 in the Supplementary Material). The custom PCB housed a microcontroller (Teensy 4.0; Fig. S1 in the Supplementary Material), a radio transceiver (NRF24L01, Fig. S1 in the Supplementary Material), and a stepper motor driver (A4988, Pololu, Las Vegas, Nevada, United States; Fig. S1 in the Supplementary Material) and was mounted on the posterior side of the 3D-printed holding structure. Figure 1(d) shows a photograph of the rotation module of the CV-guided motorized commutator. The translation stages feature the same custom PCB used in the rotation stage and have stepper motors mounted directly on the arena gantry [Figs. 1(b) and 1(c)]. The x-translation stage is equipped with two limit switches on either end to prevent the mounted components from running past the edge and for self-calibration of the stage [Fig. 1(c)].

### 2.2 Operating Modes

The CV-guided commutator can operate in two modes: passive and active. In the passive mode, the experimenter manually controls the commutator using a joystick, and the commands are wirelessly transmitted to the commutator (Fig. S2 in the Supplementary Material). In the passive mode, there is no computerized tracking of an animal. The control is solely based on video playback from cameras attached to the experiment arena. In the active mode, the commutator control is executed automatically by the computer without any input from the experimenter using location and heading angle estimates (Fig. S2 in the Supplementary Material).

### 2.3 Training and Evaluating Real-Time Position and Heading Direction Algorithms

The position and heading direction of the mice were estimated using the DLC toolbox—an open-source pose estimation and behavioral analysis toolbox.^27^ The toolbox was employed to train a model tailored to the arena’s environment that incorporates the CV-guided commutator. We trained three models, namely, DLC implemented on MobileNetV2,^28^ DLC implemented on ResNet,^29^ and a third model that used social LEAP estimates animal poses (SLEAP) implemented on U-Net.^30^^,^^31^ For comparing the real-time detection capabilities of these models, behavior videos from three different studies were used—mouse performing an active place avoidance task^32^ (n=3 mice); mice performing a spatial navigation task, Barnes maze^33^ (n=3 mice); and mice exploring an open-field arena (n=3) mice. In each behavioral assay, 50 randomly selected frames from videos lasting 15 min were manually annotated to generate a ground truth of the location of the head and base of the tail. These were compared with the estimated head position and position of the base of the tail from the three models evaluated. In each assay, estimated and actual positions of the head and tail base (manually annotated) were compared using a 10-pixel radius threshold. The estimation error was used as a metric for comparing the three models.

### 2.4 Implementation of Rotation and Translation Compensation

To compensate for the rotation of the animal, the model estimates the heading angle. This was achieved by estimating the x and y coordinates of the head of the animal and the base of the tail of the animal across sequential camera frames captured at a rate of 6 to 10 frames per second (FPS). The magnitude of frame-to-frame change in heading angle was computed using the dot product of the heading direction vectors, and the direction of the change, i.e., clockwise (CW) or counter-clockwise (CCW), was computed using the cross-product of the heading direction vectors. During operation, the cumulative change in heading direction was computed, and when the cumulative value surpassed a predefined threshold, the rotation motor in the active commutator was activated to alleviate torsional stresses in the cable. We used a threshold of 90 deg in the APA task and Barnes maze task and 225 deg in the linear maze task.

For translational compensation on the x stage, the model estimated the location of the head. Experiments were conducted when mice explored in a linear 1.2-m-long maze. The length of the maze was virtually divided into eight segments, each ∼150  cm long. For each frame of the behavioral video, the estimate of the mouse’s head location was used to determine the current segment occupied by the mouse. When mice moved between segments, the linear stage was activated to ensure the commutator was directly above the mouse. Active commutation was evaluated in the APA task and the linear maze.

### 2.5 Cortex-Wide Calcium Imaging During Active Commutation

We performed cortex-wide mesoscale calcium imaging using a miniaturized microscope, mini-mScope^1^ through a polymer transparent cranial window^34^^,^^35^ during the APA task using the CV-guided active commutator to transmit signals through the slip ring. Calcium activities were acquired at 15 FPS. The CMOS gain was set to a value of 55, and the LED voltage and current were set to 8 V and 0.8 A, respectively, for the blue LEDs. The blue LEDs were pulsed for 120 s, prior to the experiment, to allow them to warm up and reach a stable intensity. The mice were brought into the different behavioral arenas under red light and placed into an opaque cylinder at the center of the maze ∼90  s after the LEDs were turned on. The mini-mScope was attached to the mice via three interlocking magnets. At ∼120  s, the opaque cylinder was removed, marking the start of the trial.

### 2.6 Calcium Data Pre-Processing

Calcium imaging was captured under both blue light illumination and green light illumination in alternate frames. The mean pixel intensity of each frame captured by the mini-mScope was calculated, and K-means clustering was used to classify each mean pixel intensity of the video and segregate the frame captured under blue light illumination (Calcium signals) and green light illumination (reflectance signals, see Ref. 1). K-means clustering also enables identification and removal of outlier frames in the data due to large motion artifacts or irregularities in LED intensity (∼0.04% of all frames). The videos corresponding to both illumination wavelengths were then passed through a motion correction algorithm.^36^

The calcium data videos were compressed to 80% of their original size with a bilinear binning algorithm (2022b, MathWorks, Natick, Massachusetts, United States). One frame randomly selected in each trial was used to draw a mask around the imaged brain surface and exclude the background and superior sagittal sinus artery to reduce noise in the overall fluorescence over baseline fluorescence (DF/F) signal. For each mouse, the masks across all trials were averaged to generate a mouse-specific average cortex mask. The average mask was imposed across images acquired in all trials for a mouse so that the number of pixels used in each analysis remained consistent.

Each pixel within the mask was corrected for global illumination fluctuations using a correction algorithm that produces DF/F data.^37^ The DF/F data was filtered using a zero-order phase Chebyshev band-pass filter with cutoff frequencies of 0.1 and 5 Hz (2022b, MathWorks). The resulting data were then spatially filtered with a 7-pixel nearest-neighbor average using a custom MATLAB (2022b, MathWorks) script. The resulting DF/F time series for each pixel was then z-scored.

## 3 Results

### 3.1 Comparative Analysis of Pose Estimation Toolboxes and Networks

Accurately tracking animal position and heading direction across diverse experimental assays and imaging conditions is crucial for a CV-guided active commutator to reliably mitigate wire entanglement. Several open-source markerless pose estimation models^27^^,^^30^ have demonstrated capabilities in accurately tracking mice across a wide range of imaging setups and behavioral assays. To determine the model most suitable for integration with a CV-guided active commutator, we compared three models—DLC implemented in MobileNetV2,^27^^,^^28^ DLC implemented in ResNet,^27^^,^^29^ and SLEAP implemented on U-Net^30^^,^^31^—based on four criteria. First, the selected markerless pose estimation model should be able to track the position of the mouse in a wide range of imaging conditions and behavioral assays. Second, the model should be accurate and reliable such that it ensures minimal tracking errors. Third, the implementation of the model should allow for near real-time (>6  Hz) estimation of the animal’s position and heading angle, allowing up to 10 compensatory adjustments to the commutator position and angular orientation per minute. Finally, the model should be executable on a regular desktop computer without necessitating an expensive graphic processing unit, ensuring that the system is accessible and cost-effective for widespread laboratory use.

To evaluate the efficacy of the three models, we tested markerless tracking capabilities on three independent behavioral assays that can be tracked using a single overhead camera. In the APA task, mice are introduced into a circular rotation arena and receive mild foot shocks when entering a designated sector.^32^^,^^38^ As the arena rotates, mice must continuously move and adjust their position to avoid entering these shock zones.^38^ The behavioral camera encompassed a circular area of 34.5 cm diameter and had a bed of parallel wires for delivering shock. The Barnes maze task, a dry-land version of a spatial task for studying the effects of aging on navigation in rats, is an avoidance task in which rodents learn to leave the center of a large circular platform (where they are exposed under bright, aversive lights) to find an escape hole among those at the edge of the platform.^33^ Only one of the holes provides access to a nest space. The Barnes maze allowed the evaluation of real-time markerless tracking of an animal exploring a large open environment (1 m diameter). Third, we used a standard open-field arena, where mice explored the whole extent of the maze, exhibiting a variety of behaviors, including thigmotaxis, rearing, and grooming.

**Fig. 2:**
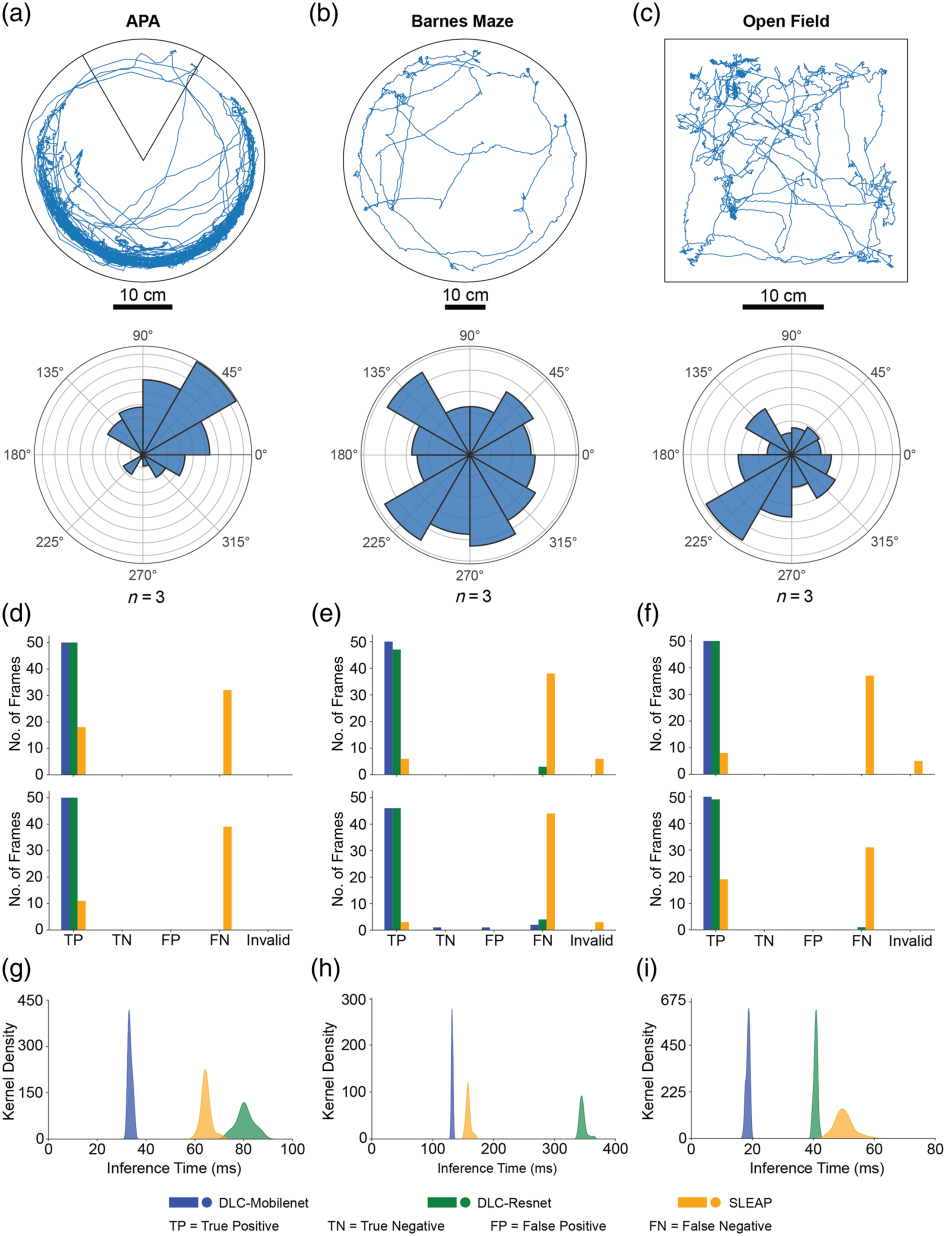
Evaluation of pose estimation toolboxes and convolution neural networks for real-time position and heading direction estimates. (a) Trajectory plot of a mouse performing the active place avoidance task^32^ (top) and polar histogram showing the distribution of the head direction (n=3 mice) in the same arena (bottom). The pose tracking was done using the DLC toolbox. (b) Trajectory plot of a mouse performing the Barnes maze task^33^ (top) and polar histogram showing the distribution of the head direction (n=3 mice) in the same arena (bottom). (c) Trajectory plot of a mouse in an open field arena (top) and polar histogram showing the distribution of the heading direction (n=3 mice) in the same arena (bottom). The pose tracking was done using the DeepLabCut toolbox. (d) Accuracy of real-time estimation of head position (top) and tailbase position (bottom) of the mouse during APA behavior for each of the three models: DLC implemented on MobileNetV2, DLC implemented on ResNet, and SLEAP implemented on U-Net. (e) Accuracy of real-time estimation of the head position (top) and tailbase position (bottom) of the mouse during Barnes maze behavior for each of the three models. (f) Accuracy of real-time estimation of the head position (top) and tailbase position (bottom) of the mouse during open field behavior for each of the three models. (g) Distribution of position inference time during APA behavior for the three models evaluated. (h) Distribution of position inference time during Barnes maze behavior for the three models evaluated. (i) Distribution of position inference time during open field behavior for the three models evaluated.

Figures 2(a)–2(c) show the results of markerless tracking of the mouse head position in each of the three behavioral assays, as well as the range of head angle estimated. As expected, in the APA task, active avoidance resulted in higher occupancy in the non-shock areas of the maze, with polarized heading angles. Barnes maze tasks resulted in animals predominantly exploring the edge of the maze, with nearly uniformly distributed heading angles. Similarly, in open-field experiments, the animal position could be estimated without any large errors or jumps in position estimates. Animals had a tethered head-borne device, and tracking was robust to variations in wired connection position within the FOV.

To evaluate the accuracy of three models—DLC implemented in MobileNetV2, DLC implemented in ResNet, and SLEAP implemented on U-Net—we compared the estimation head position [Fig. 2(d)] and estimated the base of tail position [Fig. 2(e)] to 50 randomly selected manually annotated images in each of the three behavioral assays.

Of the three models, DLC implemented in MobileNetV2 consistently demonstrated superior performance across all tasks. In the APA task, DLC implemented in MobileNetV2 achieved perfect accuracy (100% precision and sensitivity) for both head and tailbase predictions. Similarly, in the Barnes maze task, DLC implemented in MobileNetV2 achieved 100% accuracy for head prediction and 97.87% and 95.83% precision and sensitivity, respectively, for tailbase prediction. In the open field, DLC implemented in MobileNetV2 again achieved 100% accuracy for both head and tailbase predictions.

We also determined the time taken for estimation or inference time. The mean inference time for detecting head and tail position in an image frame was 61.3±50.5  ms for DLC implemented in MobileNetV2. In comparison, DLC implemented in ResNet took 155.7±135.9  ms, and SLEAP implemented in U-Net took 91.0±48.2  ms. Based on these results, we concluded that the DLC implementation in the MobileNetV2 model was ideal for real-time computer vision feedback for the motorized commutator.

#### 3.1.1 CV feedback allows active compensation of commutator position and rotation angle in response to mouse movement and heading direction

We next evaluated the ability of the CV-guided motorized commutator to actively compensate and adjust the position and angular orientation of the commutator in response to the movement of mice in the behavioral arena.

**Fig. 3:**
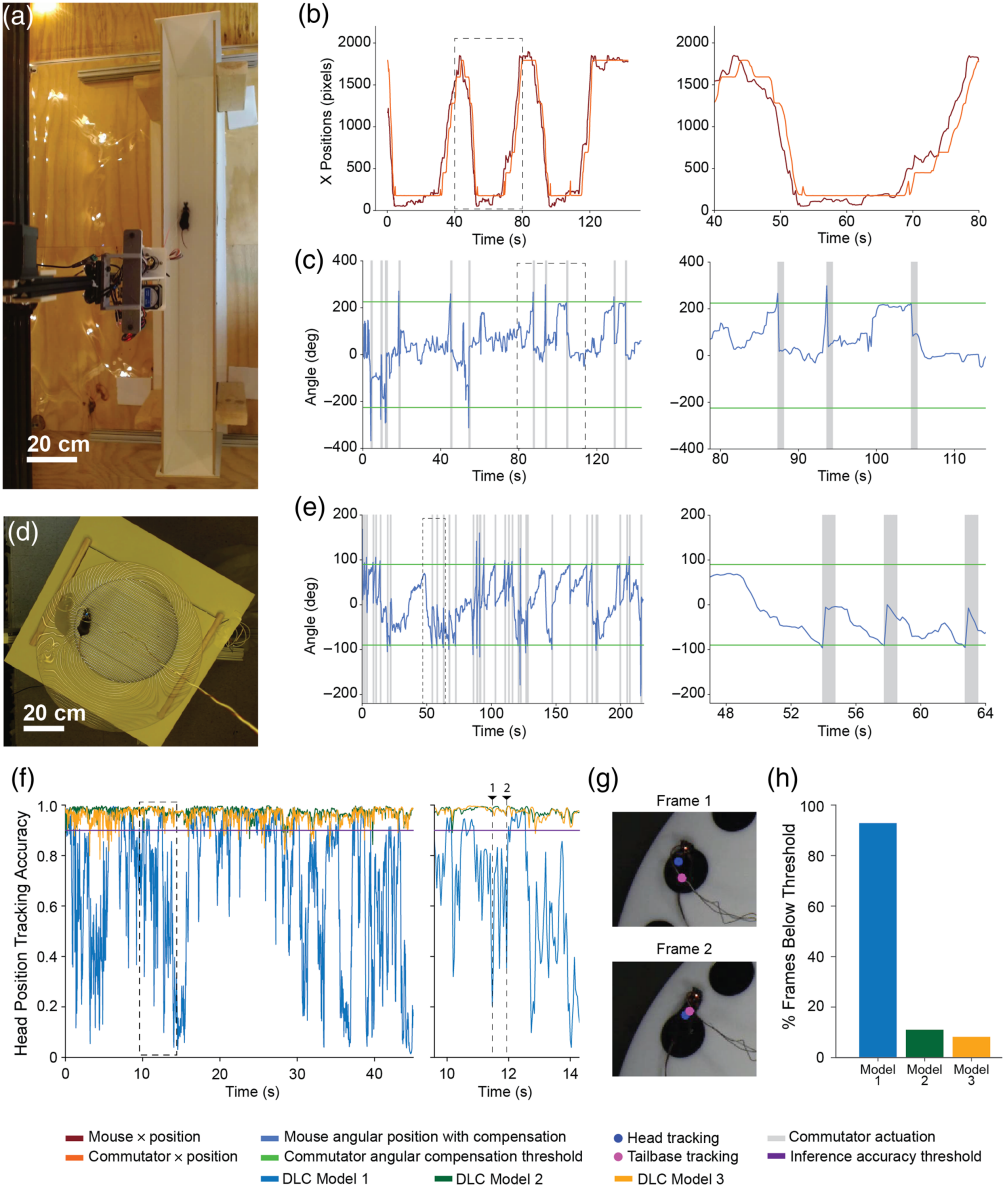
CV-guided active translation and commutation. (a) Still image captured from the overhead video camera of the mouse in a 1.2-m-long linear track arena. (b) Plot of estimated mouse position in linear track as shown in panel (a) and the position of the commutator. Left: positions over the whole trial. Right: highlighting time duration indicated in a dashed rectangle in the plot on the left. (c) Plot of estimated mouse heading direction in the linear track arena as shown in panel (a) and the angular position of the commutator. Left: angular positions over the whole trial. Right: highlighting time duration indicated in a dashed rectangle in the plot on the left. (d) Still image captured from the overhead video camera of a mouse in an active place avoidance arena. (e) Plot of estimated mouse heading direction in the active place avoidance arena as shown in panel (d) and the angular position of the commutator. Left: angular positions over the whole trial. Right: highlighting time duration indicated in a dashed rectangle in the plot on the left. (f) Plot of the head position tracking accuracy of three DLC models implemented on MobileNetV2, trained on 40, 180, and 360 labeled frames. Left: head position tracking accuracy for the first 45 s of a Barnes maze trial. Right: highlighting time duration indicated in a dashed rectangle in the plot on the left. (g) Video frames from positions 1 and 2 as indicated in panel (f) for DLC model 1 with the head and tailbase tracking points marked on the images. (h) Plot showing the percentage of tracked frames below the 90% accuracy threshold for each of the three models over a whole trial (Video 1, mp4, 2.37 MB [URL: https://doi.org/10.1117/1.NPh.11.3.034312.s1]; Video 2, mp4, 9.42 MB [URL: https://doi.org/10.1117/1.NPh.11.3.034312.s2]; Video 3, mp4, 8.10 MB [URL: https://doi.org/10.1117/1.NPh.11.3.034312.s3]; Video 4, mp4, 5.21 MB [URL: https://doi.org/10.1117/1.NPh.11.3.034312.s4]).

To assess the combined translation and rotation capabilities, mice were tracked along a long linear maze measuring 1.2 m long [Fig. 3(a), Video 1]. The length of the maze was virtually divided into eight segments, each ∼150  cm long, and the position of the commutator was adjusted when mice moved from one segment to the next. The heading angle of the commutator was also adjusted when mice accumulated heading angle exceeded a threshold.

A plot of the real-time position estimate of the mouse within the linear track and the position of the commutator directly above it is shown in Fig. 3(b). Within the same experiment, the mouse heading angle was also estimated and accumulations of cumulative head motion of 225 deg resulted in compensation of the commutator to mitigate wire entanglement [Fig. 3(c)]. We were able to reliably run trials lasting 24 min, with no errors in tracking and active compensation observed.

To assess just the rotation capabilities of the CV-guided motorized commutator, we repeated the experiment in the APA task. Mice were tracked in a circular maze of diameter 34.5 cm [Fig. 3(d), Video 2]. The heading angle of the commutator was adjusted when mice’s accumulated heading angle exceeded a threshold. A plot of the real-time heading angle estimates of the mouse within the circular track and the heading angle of the commutator directly above it is shown in Fig. 3(e). Accumulations of cumulative head motion of 90 deg resulted in compensation of the commutator to mitigate wire entanglement [Fig. 3(e)]. We were able to run n=150 trials ranging from 10 to 45 min, cumulatively 30 h of testing, with no errors in tracking and active compensation observed. The longest continuous experiment we have performed lasted 85 min. To evaluate whether longer-duration experiments with active commutation could be performed, we also conducted four consecutive trials in the APA arena without shutting down the commutator software. The CV commutator was paused when mice were switched in between trials lasting 45 min. Cumulatively, in an experimental session lasting 3.3 h, the CV commutator was able to reliably track and compensate for the rotation motion of the animals (Fig. S3 in the Supplementary Material). Videos 3 and 4 illustrate the performance of the CV commutator over a single whole 45-min trial and all four trials visualized in parallel, respectively.

One confounding factor in real-time estimation of heading direction angle or position is that the animal being tracked can be occluded by wires or other artifacts. The CV commutator accounts for this issue by only considering frames where both the head and tailbase tracking accuracy is above a threshold of 90%. Depending on the contrast between the mouse and the background, as well as the quality of training data, the numbers of frames below this accuracy threshold of 90% can vary. To assess the influence of the size of the training data on the tracking accuracy of the DLC tracking model, three DLC models implemented on MobileNetV2 were trained on different amounts of labeled data. The models were trained on 40, 180, and 360 labeled frames. Aside from the number of labeled frames, all other training parameters were kept the same. In addition, Barnes maze behavior videos were used for this evaluation because the trained DLC models for the Barnes maze performed worse than models trained on other behavioral videos—keeping the number of labeled frames the same and other training parameters.

A plot comparing the head position tracking accuracy of three DLC models trained on different numbers of labeled data is shown in Fig. 3(f). The plot shows tracking data for the first 45 s of a Barnes maze behavior video. We were able to get better tracking accuracy with increased labeled training data. Figure 3(g) shows two instances where DLC model 1 had head position tracking accuracy values below the 0.9 accuracy threshold. Similarly, a plot showing the number of frames with tracking accuracy below the accuracy threshold in the whole trial per training model is shown in Fig. 3(h). DLC model 1, trained on the least number of labeled frames, resulted in over 90% of frames below the threshold, and DLC model 3, trained on the largest number of labeled frames, resulted in 8.2% of frames below the threshold. The algorithm further requires estimation once every five frames. Thus, it is possible to ignore frames with low tracking accuracy without affecting active commutation. Overall, with a well-trained model, it is possible to limit the number of frames disregarded by the algorithm for active commutation, but this must be taken into consideration when implementing CV-guided active commutation in behavioral contexts beyond what has been tested in this work.

#### 3.1.2 Stable *in vivo* imaging during active commutation

**Fig. 4:**
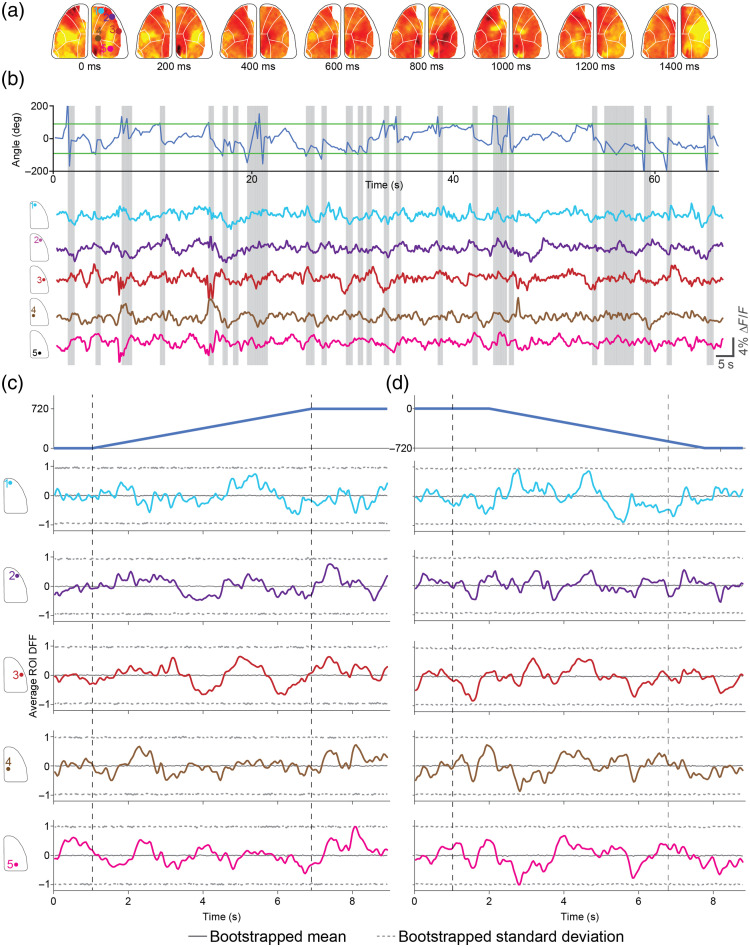
Wide-field calcium imaging in freely behaving mice during active commutation. (a) Pseudo-color DF/F z-score heat maps showing calcium activity progression during an active place avoidance trial during active commutation. (b) Top: mouse body angle tracking during a trial in the active place avoidance task. Gray lines denote active commutation periods to account for mouse angle changes greater than 90 deg, highlighted in green. Bottom: average DF/F z-score maps plotted for a wide range of regions of interest following the Allen Brain Atlas across one hemisphere of the brain. Gray lines denote active commutation periods to account for mouse angle changes >90  deg. (c) Top: peri-event time histograms for the average of 10 clockwise rotations of 720 deg with the active commutator. Bottom: peri-event time histograms for the corresponding average DF/F z-score across five regions of interest in the 10 clockwise rotations. Solid color lines indicate the average DF/F z-score for each region of interest. The gray solid line indicates the average of 1000 randomized bootstraps of the DF/F z-score data for the entire trial taken during the commutation period for each region of interest. The gray dashed line indicates the standard deviation of 1000 randomized bootstraps of the DF/F z-score data for the entire trial taken during the commutation period for each region of interest. (d) Top: peri-event time histograms for the average of 10 counterclockwise rotations of 720 deg with the active commutator. Bottom: peri-event time histograms for the corresponding average DF/F z-score across five regions of interest in the 10 counterclockwise rotations. Solid color lines indicate the average DF/F z-score for each region of interest. The gray solid line indicates the average of 1000 randomized bootstraps of the DF/F z-score data for the entire trial taken during the commutation period for each region of interest. The gray dashed line indicates the standard deviation of 1000 randomized bootstraps of the DF/F z-score data for the entire trial taken during the commutation period for each region of interest.

We performed wide-field imaging of calcium activities across the whole dorsal cortex using a miniaturized mesoscale imaging device^1^ [Fig. 4(a)] while routing the digital interlink through the motorized commutator. We were able to stably record calcium dynamics at 30 FPS with only 25 frames dropped during a recording lasting 10 min (0.13% of a total of 18,000 frames). Qualitatively, the recorded calcium activities in the regions of interest distributed throughout the cortex were similar to those acquired with the same device in previous studies.^1^^,^^33^ We recorded in total of 40 min, resulting in 130 frame drops (0.18% of 72,000 frames), again comparable to the results obtained in our previous work that used a commercial commutator. Note that the frame drop rate is a function of acquisition speed and the configuration of the data acquisition hardware, as well as the efficiency of the computer USB disk drive speed. The frame drops we measured in our motorized commutator system (JP41-119-01HW, JINPAT Electronics) are comparable to the frame drop rate we have observed when using a commercial static commutator system (Carousel Commutator 1× DHST 2× LED, Plexon Inc., Dallas, Texas, United States).^1^ Frame drops can occur even in the absence of commutation. As shown in Fig. S4 in the Supplementary Material, data loss occurring during acquisition even without a slip ring is comparable to data loss that occurs when the data are acquired through a slip ring, with and without active commutation. Our modular design can be modified to incorporate other slip rings or commutators with additional data transfer capabilities. Further users will need to pay attention to the data acquisition hardware for efficient data transfer with minimal frame losses.

The active commutator turns on the rotational motor when a threshold of 90 deg of rotation—threshold changes depending on the arena type and size—is exceeded from the starting heading direction angle. Active commutation occurs at a speed of ∼100  deg/s. Nevertheless, the active commutation itself may result in interfering with the neural imaging experiments in two ways—the untwisting of the wired data cable during active compensatory motion may result in mechanical displacement of the imaging device with respect to the skull and the brain. Second, the compensatory motion may be perceptible to the mouse and result in a neurophysiological response to the same that might result in artifacts.

Calcium imaging data analysis pipelines typically incorporate motion correction algorithms to account for mechanical displacements of the imaging device with respect to the brain. In our analysis pipeline, we used a rigid body error correction scheme that can correct for lateral displacements of the FOV.^36^ We quantified the overall changes in x and y displacement of the FOV as detected by the algorithm throughout the open field behavior trial and specifically around the CW and CCW compensatory motion epochs [Fig. 4(b)]. For the entire trial lasting 10 min, the average delta X corrections were −1.67±3.3  μm, and the average delta Y corrections were $9.04\times 10^{-4}\pm 0.11\;\mu m$. Within the compensatory rotation epochs lasting 140 s, the average delta X corrections were −2.0±3.2  μm, and delta Y corrections were $-1.00\times 10^{-3}\pm 0.06\;\mu m$, which is not visually different from the whole trial averages.

We next evaluated the calcium activity during the active compensation epochs. Wide-field imaging with the mini-mScope^1^ allows us to look at both global cortex-wide changes but also at regions of interest located at multiple sensory cortices and motor cortices. All these specific regions of interest may have neurophysiological changes in response to a perception of the compensatory motion. Figures 4(c) and 4(d) show peri-event calcium activity histograms of multiple selected regions of interest (ROIs) during CW and CCW active compensation epochs. We found that at a frame-by-frame time scale, average calcium activity is not significantly different (Bonferroni correction, 1000 random bootstraps) from the whole trial bootstrapped calcium activity traced for all the ROIs analyzed. Thus, we can conclude that active compensation does not introduce any neurophysiological artifacts.

## 4 Discussion and Conclusion

We present a CV-guided active translating commutator that can track and move along with a mouse in behavioral arenas that are large (>1  m) while minimizing cable length and using overhead behavioral cameras that simultaneously track the behavior of the animal. The commutator was tested extensively in three separate behavioral experiments—where mice were tracked over large arenas (Barnes maze), in standard open-field environments where most of the natural repertoire of behaviors were recapitulated, and the active place avoidance tasks where mice performing rapid movements were reliably tracked in real-time over a shocking grid background. We found that the model using the DLC algorithm implemented in MobileNetV2 performed reliably, with 100% precision and sensitivity in the APA task, 98.94% precision and 97.12% sensitivity in the Barnes maze task, and 100% precision and sensitivity in the open field. Importantly, the model had an inference time of 61.3±50.5  ms when executed on a standard desktop PC. Further, data acquisition using miniaturized microscopes was performed with the same computer. Thus, this is an approach that can be implemented in a wide variety of behavioral experiments using minimal computational resources. In summary, we show that active commutation can be achieved without errors in 24 min of testing in a linear track and over 30 h of testing in the APA task.

The CV-guided active commutator uses inexpensive open-source hardware elements for translation and rotation of the slip ring. The linear actuator and the rotary actuators both use the OpenBuilds stepper motors and gantries, which are modular, and can be adapted for much larger arenas. Although we implemented translation in one direction in this study, in the future, the approach can be extended to translation in two directions. Thus, miniaturized devices with short lengths (<1  m) wired tethered can be used to interface with head-borne devices when mice are exploring significantly larger arenas. In comparison with existing active commutation approaches, our approach simplifies the experiment but does not require any modifications to the head-borne imaging and recording devices. Thus, the approach is compatible with most head-borne devices that are already being used by neuroscience laboratories.

Active commutation could be a useful extension to existing commutators. First, it will allow longer experiments without the need for experimenter intervention. This may be critical for experiments where experimenter intervention might result in behavioral artifacts. Second, CV-commutation may open up new experimental capabilities. For instance, newly developed miniaturized two-photon^2^ (2P) and three-photon^39^ (3P) miniaturized microscopes rely on significant engineering efforts to reduce the torsional integrity of the optic fibers used for light delivery. Indeed, there have been active efforts to design miniaturized 2P microscopes that utilize fibers with reduced torsional integrity for light delivery.^40^ With the ability to actively commutate, it may be possible to relax this design constraint and design higher-performance 2P and 3P miniaturized microscopes that do not require ultra-thin optic fibers. Further, arrays of multiple imaging systems could be developed, as we have recently demonstrated,^41^ where active commutation is a necessity.

Although we strived to test the CV-guided active commutator in several behavioral contexts, it is possible that implementing this in behavioral assays where mice need to be tracked in arenas offering low contrast may result in lesser accuracy and precision in estimating position and heading direction. Although the algorithm accounts for instances when tracking accuracy is diminished, this strategy can only work if the animal heading direction can be tracked reliably in most of the image frames. This is an important consideration when designing experiments using the CV commutator. We incorporated a manual override option to mitigate this issue. Second, the issue may potentially be mitigated by imaging the mice from the bottom of the arena if compatible with the behavioral test. Finally, we note that robotic actuation of the commutator, particularly translation, may be perceived as the sweeping of an aerial predator, which might result in innate defensive behaviors by the mice. Although we did not observe this in our experiments, this is an issue that might arise in other experimental contexts.

## Supplementary Material











## Data Availability

All computer-aided design (CAD) files, code, and software associated with this paper are available at https://github.com/bsbrl/Motorized-Commutator.git.
