# Supplementary material for: Computer vision–guided open-source active commutator for neural imaging in freely behaving animals
Source: Neurophotonics. 2024 Sep 26;11(3):034312. doi: 10.1117/1.NPh.11.3.034312 (PMC11425088; doi:10.1117/1.NPh.11.3.034312)
Supplement: Supplementary file 1 [file NPh_011_034312_SD001.pdf]

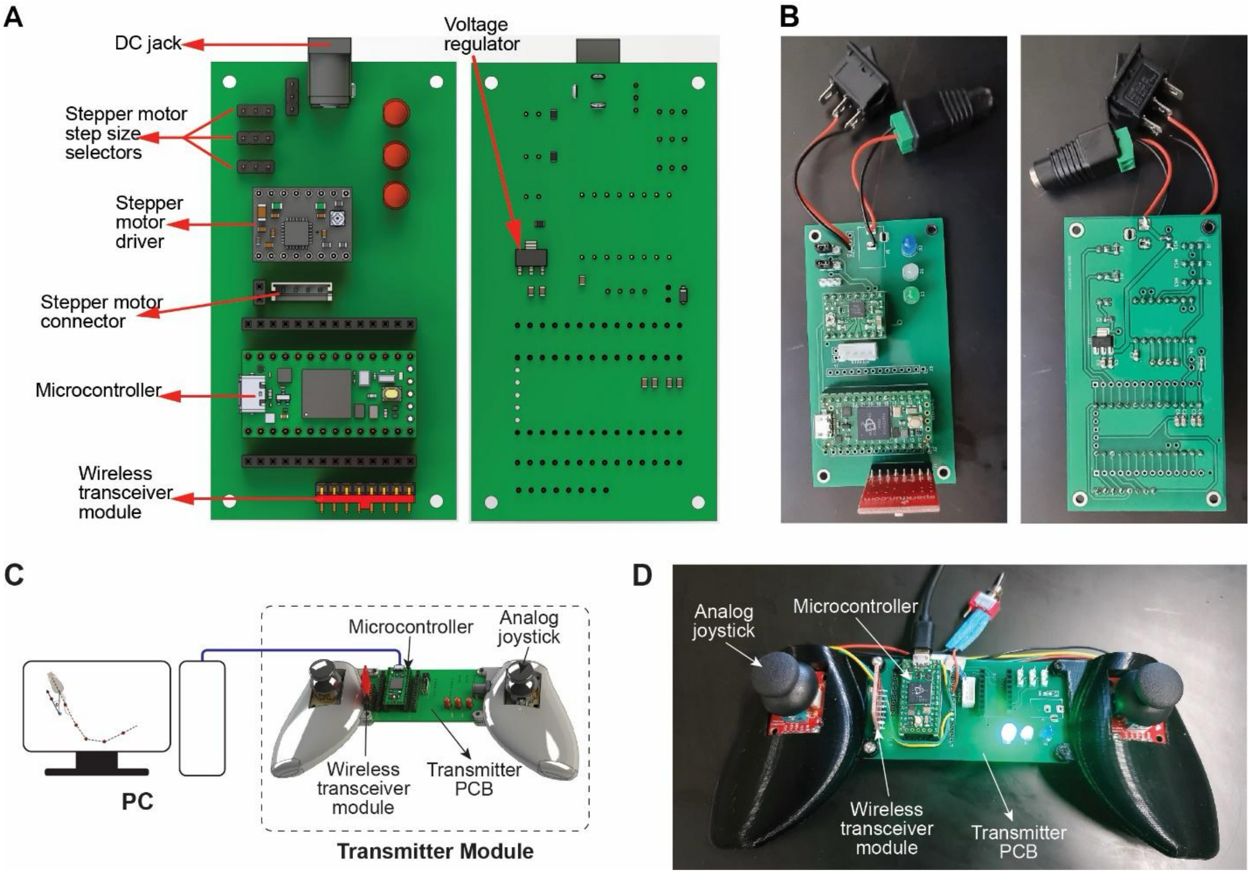

Figure S1. Active motorized commutator PCB. (A) Detailed top and bottom view of the motorized commutator PCB. (B) Top and bottom photo of a fully assembled motorized commutator PCB. (C) This panel illustrates the schematic connections between the personal computer (PC) and the transmitter module within the system. In the active operation mode, the transmitter module receives movement commands from the computer vision tracking algorithm and transmits these commands wirelessly to the motorized commutator and/or gantry in the experiment arena. In the passive operation mode, the transmitter module only gets power from the PC and control of the motorized commutator is done through the joysticks. Mode transition is enabled by the actuation of a toggle switch. (D) Photo of the transmitter module.

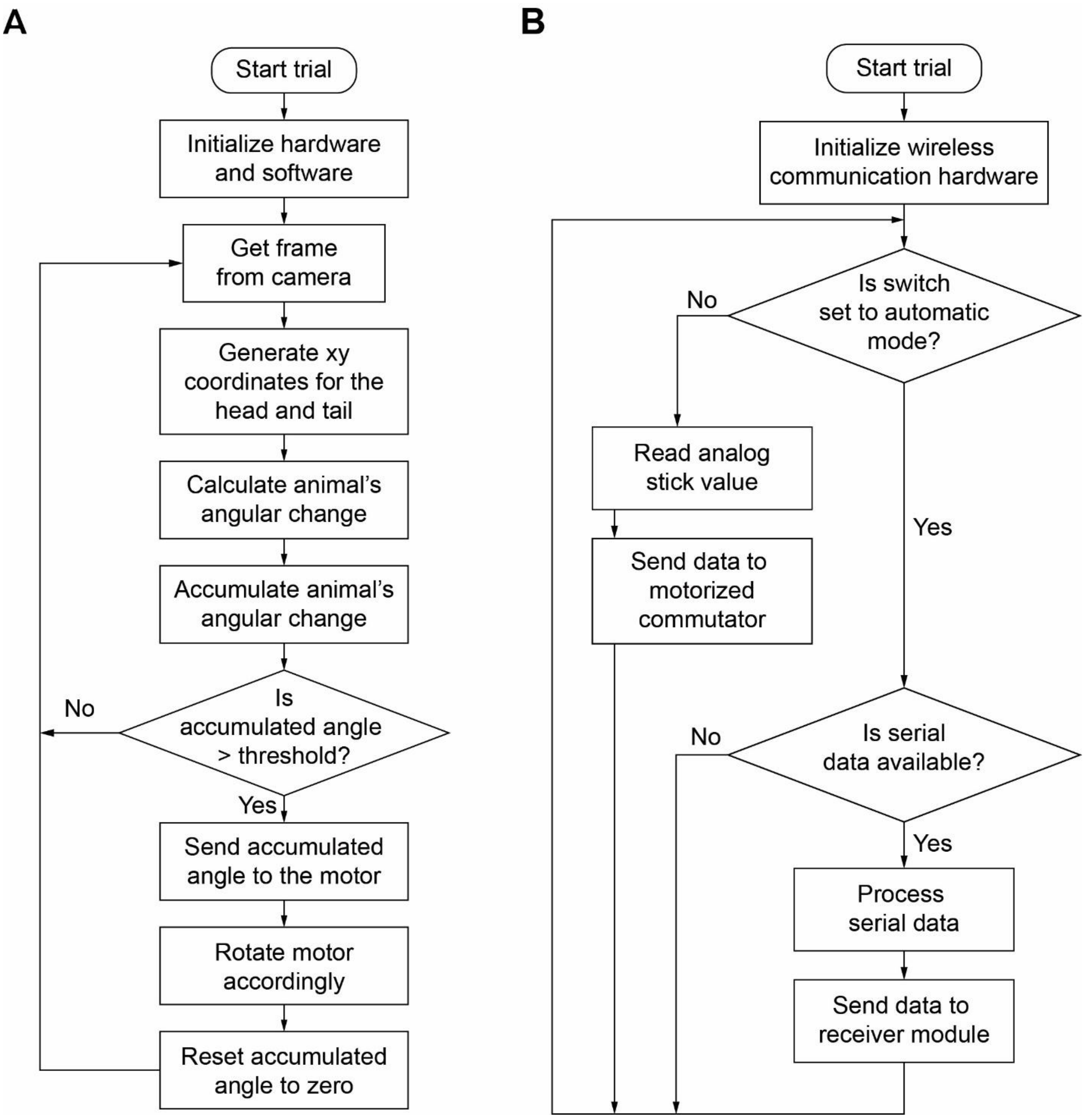

Figure S2. **Schematic representation of the cv guided active commutator control algorithm.** (A) System flow chart showing how the cv guided active commutator algorithm works. (B) System flow chart showing how the software on the transmitter module interacts with the cv guided active commutator algorithm and the commutator hardware.

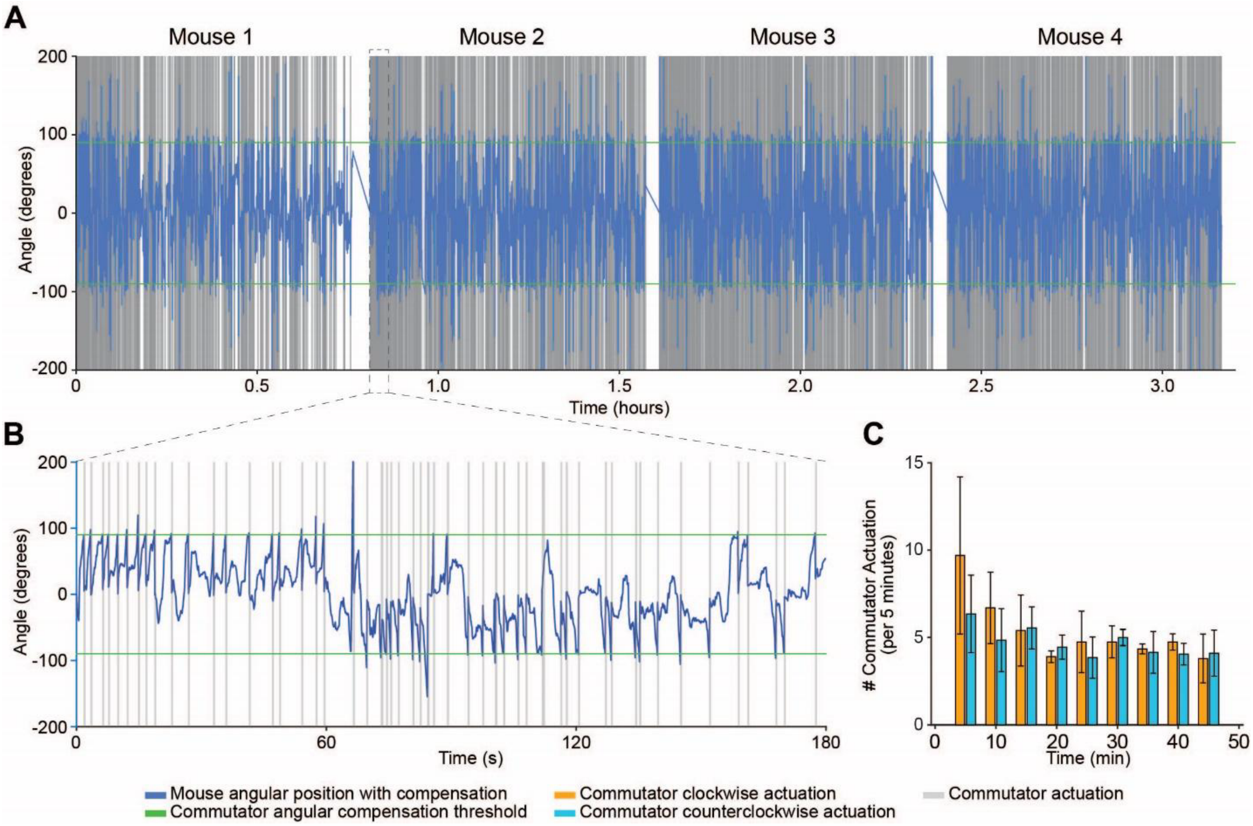

Figure S3. Longitudinal recordings using the active commutator across different mice. (A) Longitudinal recordings using the active commutator in the active place avoidance task for over 3 hours. Four 45-minute trials were conducted across four independent mice with a pause in-between each mouse to clean the arena. (B) Zoomed in view of a 3-minute portion of commutation from (A). (C) Average clockwise and counterclockwise actuation in each 45-minute trial. Error bars indicate standard deviation.

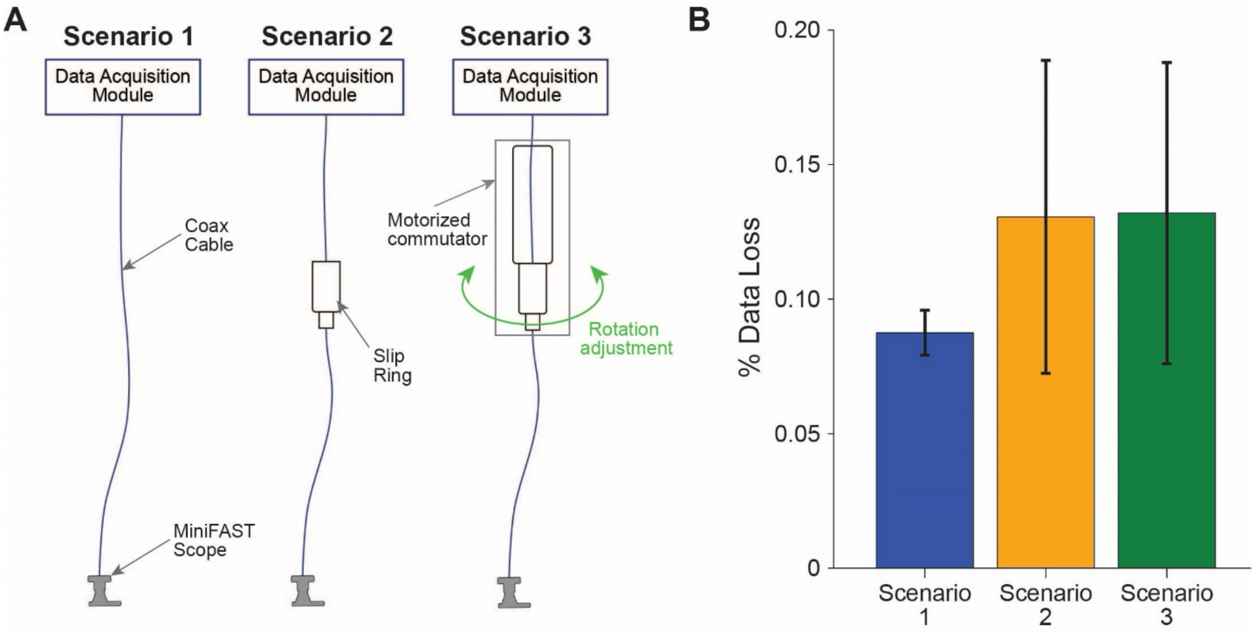

Figure S4. Testing data transmission loss through a coaxial slip ring with active commutation. (A) Testing data transmission loss through the coaxial slip ring in three scenarios, with no slip ring, slip ring with no movement, and slip ring with active commutation. (B) Data transmission loss from 10 min experiments recorded at 30 FPS, (No significant difference One way ANOVA $F(2,9)=1.16$, $p<0.35$). Error bars indicate standard deviation.

Table S1. Features of CV-guided active commutator and other motorized commutators. An overview of the features of currently published motorized commutators. Sensing approach: the method used to detect animal's change in heading angle and position. Active sensing: direct detection of animal's change in heading angle and position without relying on cable feedback. Bandwidth sharing: indicates whether the system necessitates sharing neural data bandwidth for acquiring animal pose information. Adaptability to multiple recording modalities: assesses the system's compatibility with various neural recording techniques.

**Motorized commutator**	**Sensing approach**	**Active sensing**	**Bandwidth sharing**	**Adaptability to multiple recording modalities**
Fee & Leonardo, 2001	Torque sensor	No	No	Low
Roh et al., 2015	Video-based	No	No	Low
Barbera et al., 2020	Magnetic rotation sensor	No	No	High
Jost-Mousseau et al., 2023	Inertial Measurement Unit	Yes	Yes	Low
Newman et al., 2023	Inertial Measurement Unit	Yes	Yes	Medium
Corte et al., 2024	Hall-effect sensor	No	No	Low
CV-guided active commutator (This work)	Video-based	Yes	No	High
